# Losing the stem-loop structure from metazoan mitochondrial tRNAs and co-evolution of interacting factors

**DOI:** 10.3389/fgene.2014.00109

**Published:** 2014-05-01

**Authors:** Yoh-ichi Watanabe, Takuma Suematsu, Takashi Ohtsuki

**Affiliations:** ^1^Department of Biomedical Chemistry, Graduate School of Medicine, The University of TokyoTokyo, Japan; ^2^Department of Biotechnology, Okayama UniversityOkayama, Japan

**Keywords:** mitochondrial tRNA, D-arm, T-arm, tRNA nucleotidyltransferase, aminoacyl-tRNA synthetase, elongation factor Tu, ribosome

## Abstract

Conventional tRNAs have highly conserved sequences, four-armed cloverleaf secondary structures, and L-shaped tertiary structures. However, metazoan mitochondrial tRNAs contain several exceptional structures. Almost all tRNAs^Ser^ for AGY/N codons lack the D-arm. Furthermore, in some nematodes, no four-armed cloverleaf-type tRNAs are present: two tRNAs^Ser^ without the D-arm and 20 tRNAs without the T-arm are found. Previously, we showed that in nematode mitochondria, an extra elongation factor Tu (EF-Tu) has evolved to support interaction with tRNAs lacking the T-arm, which interact with C-terminal domain 3 in conventional EF-Tu. Recent mitochondrial genome analyses have suggested that in metazoan lineages other than nematodes, tRNAs without the T-arm are present. Furthermore, even more simplified tRNAs are predicted in some lineages. In this review, we discuss mitochondrial tRNAs with divergent structures, as well as protein factors, including EF-Tu, that support the function of truncated metazoan mitochondrial tRNAs.

## INTRODUCTION

**FIGURE 1:**
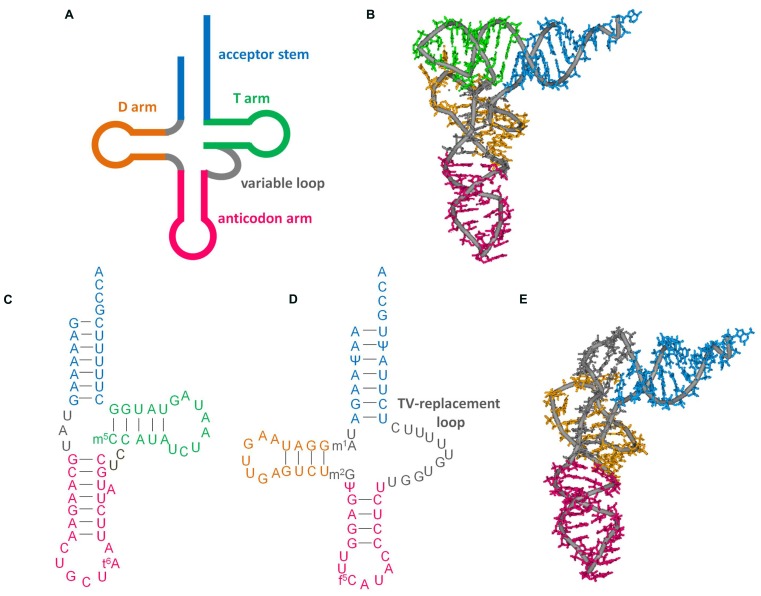
**Secondary and tertiary structures of tRNAs. (A)** Cloverleaf tRNA. **(B)** L-shape tertiary structure of cloverleaf tRNA (*Saccharomyces cerevisiae* tRNA^Phe^, PDB code 6TNA). **(C)** D-arm-lacking tRNA (bovine mt tRNA^Ser^(GCU), accession number: X15132. Modification data from tRNAdb (Jühling et al., 2009), tRNAdb ID: tdbR0000402). **(D)** T-arm-lacking tRNA (*Ascaris suum* mt tRNA^Met^, accession number: D28746; Watanabe et al., 1994; Sakurai et al., 2005b). **(E)** L-shape model of T-arm-lacking tRNA (Ohtsuki et al., 1998) built with reference to the crystal structure of yeast tRNA^Phe^. This model was built from computational analysis including molecular dynamics calculations to arrange the base locations according to tertiary interactions deduced from NMR observations (Ohtsuki et al., 1998), sequence alignment (Wolstenholme et al., 1994), and structural probing experiments (Watanabe et al., 1994).

As discussed in other articles in this special issue, conventional tRNAs are highly conserved: they have a four-armed cloverleaf secondary structure and L-shaped tertiary structure (**Figures 1A,B**; Jühling et al., 2009). However, some tRNAs encoded in mitochondrial DNA, particularly in metazoan (multi-cellular animal) mitochondria, have diverged from standard form tRNAs in a variety of ways. In this review, we focus on mitochondrial tRNAs (mt tRNAs) lacking either the dihydrouridine arm (D-arm) or the ribothimidine arm (T-arm; **Figures 1C,D**). The function of tRNA is to help decode mRNA into protein. tRNA collaborates with a variety of proteins from post-transcription to decoding in ribosomes. The unique characteristics of factors interacting with such shrunken tRNAs have been uncovered over the past several decades. In this review, these factors and their evolution will also be discussed.

## SHRUNKEN mt tRNAs

### tRNAs LACKING THE D-arm

In the 1970s, D-arm-lacking tRNAs were at first identified as non-tRNA molecules (a putative equivalent to cytoplasmic 5S ribosomal RNA) because of their short length (Dubin and Friend, 1972; Dubin et al., 1974; Baer and Dubin, 1980). Since the identification of genes in mitochondrial DNA, these truncated tRNAs have been proposed to be functional (Arcari and Brownlee, 1980; de Bruijn et al., 1980). Virtually all metazoan mitochondria have at least one of D-arm-lacking tRNA, namely tRNA^Ser^(GCU/UCU) for AGY or AGN codons (**Figure 1C**; Jühling et al., 2009). In addition, some animal mitochondria have additional D-arm-lacking tRNAs, such as tRNA^Ser^(UGA) in chromadorean nematodes (Okimoto et al., 1992), and tRNA^Cys^ in some vertebrates (Seutin et al., 1994).

The secondary structures of D-arm-lacking tRNAs have been classified into several groups based on the base pairs in T and anticodon stems (Steinberg et al., 1994). Experimental verifications of the secondary and tertiary structures have been performed using chemical modification, limited enzymatic digestion, nuclear magnetic resonance (NMR) spectroscopy, and native gel electrophoresis (de Bruijn and Klug, 1983; Ueda et al., 1985; Hayashi et al., 1997; Frazer-Abel and Hagerman, 1999, 2004; Ohtsuki et al., 2002a). Although these results support the coaxial stacking of T and acceptor stems (Frazer-Abel and Hagerman, 2008), this idea is somewhat controversial, possibly because of the structural flexibility of the D-arm-lacking tRNAs themselves (Frazer-Abel and Hagerman, 2008). The shortest possible D-arm-lacking tRNA was suggested to be 54 nt long (Steinberg et al., 1994).

Aminoacylation and EF-Tu binding of D-arm-lacking tRNAs have been demonstrated (Ueda et al., 1985, 1992; Yokogawa et al., 1989, 2000; Kumazawa et al., 1991; Watanabe et al., 1994; Hanada et al., 2001; Shimada et al., 2001; Ohtsuki et al., 2002a; Chimnaronk et al., 2005; Suematsu et al., 2005). Translation with an unmodified D-arm-lacking mammalian mt tRNA^Ser^(GCU) derivative with a GAA anticodon has been investigated using a cell-free system (Hanada et al., 2001). The ability to form a ternary complex with EF-Tu/GTP of the tRNA^Ser^(GCU) derivative is similar to that of the tRNA^Ser^(UGA) derivative, which has a four-armed cloverleaf secondary structure (Hanada et al., 2001). However, the amount of peptides produced using tRNA^Ser^(GCU) derivative is lower than that produced using tRNA^Ser^(UGA) derivative (Hanada et al., 2001).

### tRNAs LACKING THE T-arm

T-arm-lacking tRNA genes were identified in nematode mitochondria in Wolstenholme et al. (1987). Since then, T-arm-lacking tRNA genes have also been found in mitochondrial DNA in other lineages of animals, such as Arthropoda (Masta, 2000). They have a TV replacement loop instead of a variable loop and T-arm (**Figure 1D**). Isolation of nematode mt T-arm-lacking tRNAs has been performed with preparative gel electrophoresis and/or solid-phase DNA affinity purification (Watanabe et al., 1994, 1997, Ohtsuki et al., 1998; Sakurai et al., 2005a,b).

Basically, intramolecular interactions in T-arm-lacking tRNAs are thought to be identical to those in conventional tRNAs, except for interactions between T- and D- arms, because of conservation around the D-arm and the similarity of the 5′ region in the TV replacement loop to the variable loop region (Watanabe et al., 1994; Wolstenholme et al., 1994). As an analog of cloverleaf-type tRNA, an L-shape-like structure of T-arm-lacking tRNAs has been proposed (Watanabe et al., 1994; Wolstenholme et al., 1994). Hypothesized interactions have been supported by chemical and enzymatic probing and NMR spectroscopy (**Figure 1E**; Watanabe et al., 1994; Ohtsuki et al., 1998).

The aminoacylation capacity of T-arm-lacking nematode mt tRNAs has been demonstrated with mt extract or recombinant enzymes (Watanabe et al., 1994; Ohtsuki et al., 1996; Chihade et al., 1998; Lovato et al., 2001; Sakurai et al., 2005b; Arita et al., 2006). Furthermore, the formation of a tertiary complex of a T-arm-lacking aminoacyl tRNA/EF-Tu/GTP has been detected (Ohtsuki et al., 2001; Arita et al., 2006).

In nematode mt T-arm-lacking tRNAs sequenced at the RNA level, the 1-methyladenosine at position 9 is strictly conserved (Watanabe et al., 1994, 1997; Sakurai et al., 2005a,b; see also Ohtsuki and Watanabe, 2007). This modification helps maintain the tertiary structure of the tRNA, and also aids in efficient aminoacylation and formation of the ternary complex with EF-Tu/GTP (Sakurai et al., 2005b).

### tRNAs POTENTIALLY LACKING BOTH D- AND T-arms

As mentioned above, the shortest biochemically characterized tRNA is a 54-nt long mt tRNA^Ser^(UCU) from the nematode *Ascaris suum* (Watanabe et al., 1994). A computational survey of mitochondrial tRNA genes predicted the presence of tRNA genes lacking both D- and T-arms (Jühling et al., 2012a,b). More recently, after the submission of the abstract of this review, RT-PCR analyses using 5′- and 3′-RACE showed that such putative tRNA genes are indeed transcribed, and the transcripts even have a 3′CCA sequence in the nematode *Romanomermis culicivorax* (Wende et al., 2014). Note that some tRNAs are imported from the cytoplasm into the mitochondria in some animals (Rubio et al., 2008), suggesting that imported tRNAs may function in place of mitochondrial-encoded putative tRNAs lacking both D- and T-arms. Thus, functional analysis of such extremely truncated putative tRNAs is critical.

## FACTORS INTERACTING WITH tRNAs LACKING D- OR T-arms

### AMINOACYL tRNA SYNTHETASES

Aminoacyl tRNA synthetases recognize a cognate tRNA and add an aminoacyl moiety to its 3′ end. The major recognition sites of the enzymes in tRNAs are the anticodon, a discriminator base at position 73, and the acceptor stem (Giegé et al., 1998). In fact, in the case of alanyl-tRNA synthetases, even the mitochondrial enzyme uses the acceptor stem as a major recognition site (Chihade et al., 1998). However, some of the enzymes also use the D-arm in tRNA [e.g., *Escherichia coli* isoleucyl-tRNA synthetase (Nureki et al., 1994)]. Whether the mitochondrial counterparts of such enzymes encoded in nuclear genome still use D-arms as recognition sites is an interesting issue yet to be investigated. If so, even if a tRNA lost its T-arm, the enzyme could still add the aminoacyl moiety to the shrunken tRNA.

**FIGURE 2:**
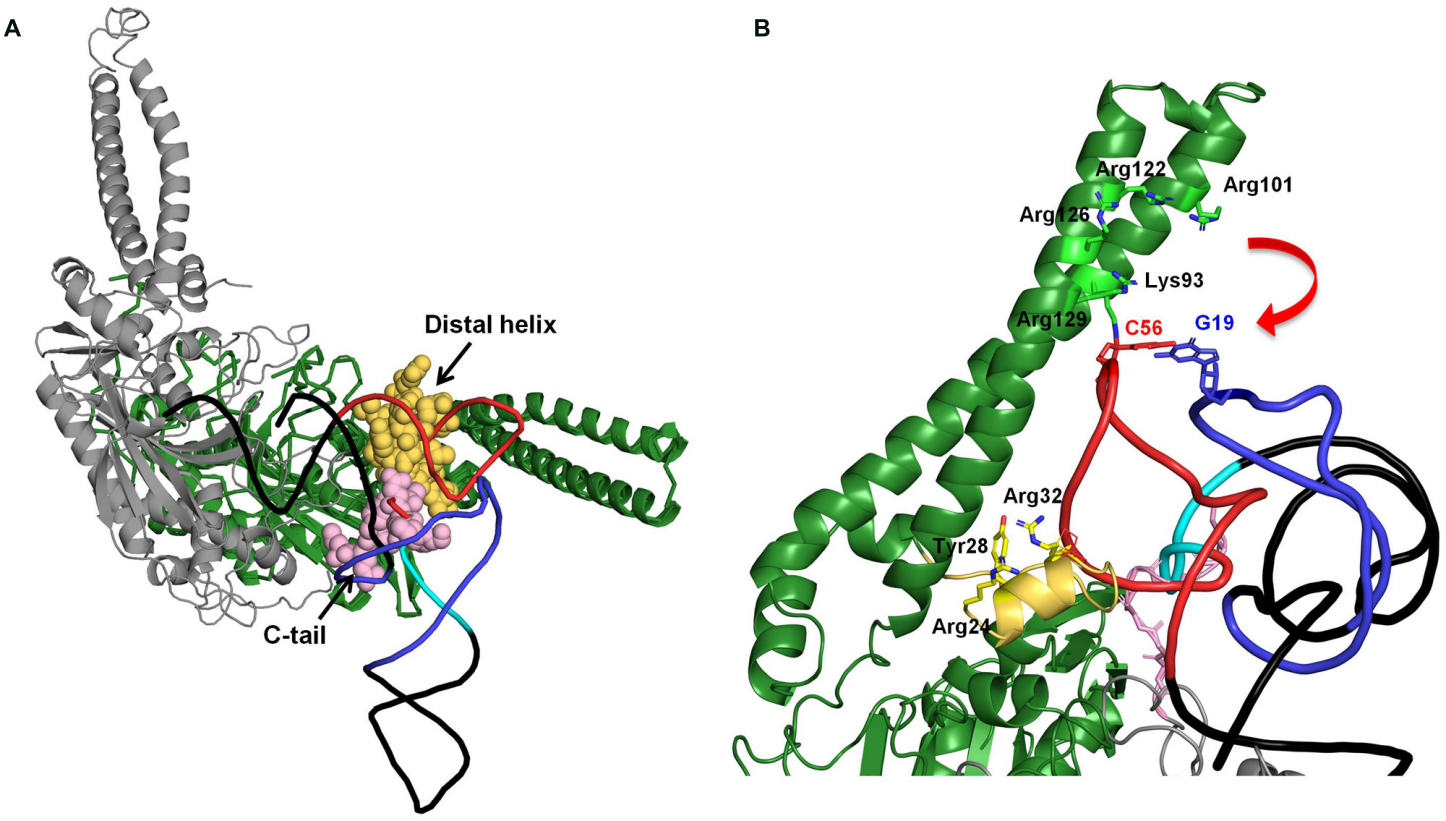
**(A)** Docking model of mammalian mt SerRS and yeast tRNA^Phe^ (Chimnaronk et al., 2005). Two subunits of the enzyme are shown in green (monomer 1), and gray (monomer 2), respectively. Distal helix of monomer 1 and C-tail of monomer 2 which are mitochondrial-specific extensions and possibly interact with the tRNA, are shown in yellow and pink, respectively. In the tRNA, D-arm, variable loop, and T-arm are shown in purple, sky blue, and red, respectively. **(B)** Putative interactions between the N-terminal coiled–coil region and the C-tail distal helix with the T-arm of the tRNA. The interactions are inferred by mutational analysis of the enzyme (Chimnaronk et al., 2005). Residues in N-terminal coiled–coil region and distal helix involved in the interaction with tRNA are shown in stick model (Chimnaronk et al., 2005).

On the other hand, seryl-tRNA synthetase (SerRS) uses recognition sites other than the anticodon and acceptor stem. Bacterial SerRS recognizes T- and characteristic long variable arms in bacterial tRNA^Ser^ (Asahara et al., 1994) using the N-terminal coiled–coil region (Biou et al., 1994). However, metazoan mt tRNA^Ser^ has lost its long variable arm, and even the D-arm is absent in tRNA^Ser^(GCU/UCU). Thus, how mt SerRS recognizes mt tRNA^Ser^ without its long variable arm is an interesting question. Earlier studies suggested that mammalian mt SerRS can recognize not only mt tRNA^Ser^ but also bacterial tRNA^Ser^; however, bacterial SerRS could not recognize mt tRNA^Ser^ (Kumazawa et al., 1991). Also, mammalian mt SerRS recognizes the T-loop of both D-arm-lacking tRNA^Ser^ and cloverleaf-type tRNA^Ser^(UGA) without the long variable arm, and further requires a T-loop/D-loop interaction with tRNA^Ser^(UGA) (dual-mode recognition; Ueda et al., 1992; Shimada et al., 2001). The crystal structure of a mammalian mt SerRS, a model of it complexed with tRNA, and mutational analyses suggest that the N-terminal coiled–coil region, the distal helix, and the C-tail interact with the T-arm of mt tRNA^Ser^ (Chimnaronk et al., 2005, **Figure 2**). Mutational analysis of mammalian mt SerRS showed the substitution of some of the residues in N-terminal coiled–coil region (shown in stick model in **Figure 2B**) reduced the aminoacylation activities of either one of two mt tRNAs^Ser^ or both, suggesting that interaction of these residues with the tRNA in the enzyme-tRNA complex (Chimnaronk et al., 2005, **Figure 2B**). To maintain these interactions, the movement of N-terminal coiled–coil region (shown as a red arrow) is expected (Chimnaronk et al., 2005, **Figure 2B**). Furthermore, mutational analysis of the enzyme suggested that, for the recognition of tRNA^Ser^(UGA) which have T-loop/D-loop interaction, Arg24, Tyr28 and Arg32, in the distal helix and the Lys93 and Arg122 on the N-terminal coiled–coil region are important (**Figure 2B**). On the other hand, for the D-arm-lacking tRNA^Ser^(GCU), Arg24 and Arg32 flanking the distal helix, and Arg129 on the N-terminal helical region are crucial (**Figure 2B**). Thus, with the dual-mode recognition, mammalian mt SerRS recognizes two tRNA^Ser^ isoacceptors which have different secondary structures using distinct sets of the residues.

Interestingly, in chromadorean nematode mt, two tRNAs^Ser^ have lost their D-arms, but the remaining 20 tRNAs have lost their T-arms. It is of interest whether nematode mt SerRS also recognize the T-arm. If so, that may explain why only tRNAs^Ser^ have kept their T-arms in nematode mitochondria. However, it seems reasonable that the secondary structure of tRNA is governed by EF-Tu and ribosomes rather than aminoacyl-tRNA synthetase (ARS) during evolution, because the recognition mode of each ARS is constrained by only one or a few tRNA, while that of EF-Tu or the ribosome is constrained by over 20 tRNAs.

### EF-Tu

**FIGURE 3:**
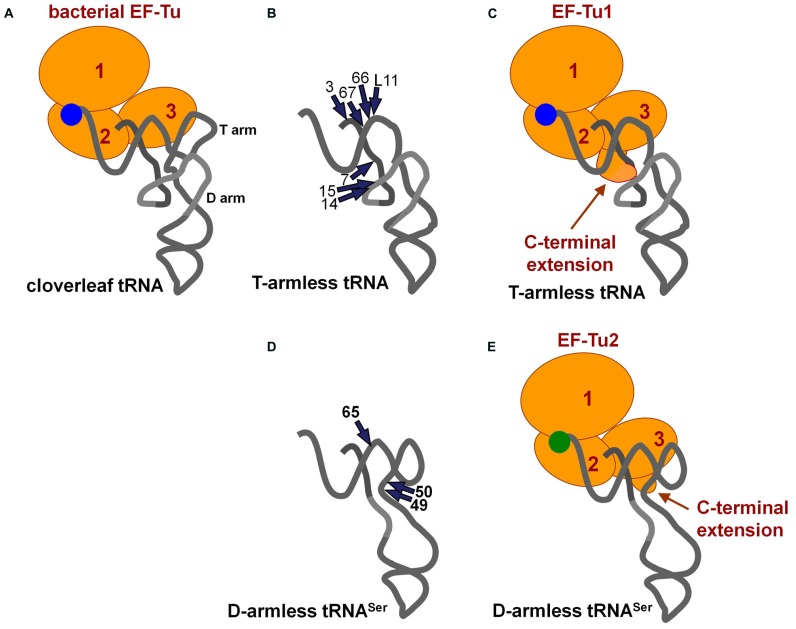
**(A)** Ternary complex of conventional tRNA/EF-Tu/GTP (Nissen et al., 1995). **(B)** Summary of ethylnitrosourea (ENU) modification interference studies of T-arm-lacking mt tRNA with *C. elegans* mt EF-Tu1 (Sakurai et al., 2006). The phosphate groups important for EF-Tu1 binding are shown with arrows. **(C)** A model of the ternary complex of T-arm-lacking tRNA/mt EF-Tu1/GTP (Sakurai et al., 2006). The model was based on the structure shown in **(A)** and in **Figure 1E**. A possible interaction between the C-terminal extension of EF-Tu1 and the region around position 9 in T-arm-lacking tRNA is suggested by modification interference as summarized in **(B)**, cross-linking studies, and the tRNA binding activity of the C-terminal deletion mutants of EF-Tu1 (Ohtsuki et al., 2001; Sakurai et al., 2006). **(D)** Summary of ENU modification interference study of D-arm-lacking mt tRNA with *C. elegans* mt EF-Tu2 (Suematsu et al., 2005). The tRNA structure was inferred from the model proposed by Steinberg and his co-workers (Steinberg et al., 1994). The phosphate groups important for EF-Tu2 binding are shown by arrows. **(E)** A model of the ternary complex of D-arm-lacking mt tRNA^Ser^/*C. elegans* mt EF-Tu2/GTP (Suematsu et al., 2005). A possible interaction between the C-terminal extension of EF-Tu2 and acceptor-T helix in D-arm-lacking tRNA is suggested by modification interference study as summarized in **(D)**, binding assays of mutant tRNAs, and the binding activity of EF-Tu mutants with C-terminal deletions (Suematsu et al., 2005).

The EF-Tu/GTP complex delivers aminoacyl-tRNAs to the A-site in ribosomes. Bacterial EF-Tu binds to the aminoacyl-moiety, a part of acceptor stem, and the T-arm (Nissen et al., 1995; **Figure 3A**), and it cannot bind to a tRNA analog missing the T-arm (Rudinger et al., 1994). In mitochondria, nuclear-encoded EF-Tu exists. Due to the presence of aminoacyl-tRNAs missing the T-arm in nematode mitochondria (Watanabe et al., 1994; Ohtsuki et al., 1996), the EF-Tu counterpart in nematode mitochondria should use an alternative binding mode for T-arm-lacking tRNAs. In fact, nematode mitochondria have two EF-Tu homologs, and only one of them (EF-Tu1) binds to T-arm-lacking tRNAs (Ohtsuki et al., 2001; Arita et al., 2006). *Caenorhabditis elegans* mt EF-Tu1 has an approximately 60 amino-acid extension at the C-terminus (domain 3′) that is essential for binding to T-arm-lacking tRNAs (Ohtsuki et al., 2001; Sakurai et al., 2006). The extension likely interacts with the D-arm region of T-arm-lacking tRNAs through positive charges in Lys residues (Sakurai et al., 2006; **Figures 3B,C**). Interestingly, *C. elegans* mt EF-Tu1 lacks binding ability to cloverleaf-type tRNAs (Ohtsuki et al., 2001), which are missing in *C. elegans* mitochondria (Okimoto et al., 1992). In another lineage of nematode, *Trichinella*, mitochondria have T-arm-lacking tRNAs, cloverleaf-type tRNAs, and D-arm-lacking tRNAs^Ser^ (Lavrov and Brown, 2001). *Trichinella* also have two EF-Tu homologs, and one of them (EF-Tu1) binds to both T-arm-lacking tRNAs and cloverleaf-type tRNAs (Arita et al., 2006). Interestingly, *Trichinella* mt EF-Tu1 has a 41-residue C-terminal extension shorter than that in *C. elegans* mitochondria (Arita et al., 2006). A mutant of *C. elegans* mt EF-Tu1 with a 13-residue deletion at the C-terminus (43-residue extension left) cannot bind to T-arm-lacking tRNAs (Sakurai et al., 2006). Although the detailed tRNA binding mode of *Trichinella* mt EF-Tu1 has not been elucidated, it could be similar but not identical to that of *C. elegans* mt EF-Tu1. Note that *C. elegans* EF-Tu1 binds to only T-arm-lacking tRNAs, while *Trichinella* EF-Tu1 binds to T-arm-lacking tRNA, D-arm-lacking tRNA, and cloverleaf tRNA (Ohtsuki et al., 2001; Arita et al., 2006).

Nematode mitochondria have another EF-Tu homolog, EF-Tu2. Nematode mt EF-Tu2 has a short (about 15-residue) C-terminal extension, and it binds to D-arm-lacking tRNA^Ser^, but not to T-arm-lacking or cloverleaf-type tRNAs (Ohtsuki et al., 2001; Suematsu et al., 2005; Arita et al., 2006). *C. elegans* mt EF-Tu2 binds to a region of the T-arm exposed due to the missing interaction between the T-arm and D-arm (**Figures 3D,E**; Suematsu et al., 2005). Interestingly, D-arm-lacking tRNAs in this species have anticodons for serine, and EF-Tu2 binds only to Ser-tRNA and accepts neither Ala-tRNA nor Val-tRNA with the same backbone (Ohtsuki et al., 2002b; Arita et al., 2006). This is likely due to the evolution of the aminoacyl-moiety binding pocket in EF-Tu2 to specialize in binding with the seryl moiety because of a unique adaptation in Ser-tRNA (Sato et al., 2006).

More recently, in some taxa other than the nematodes, such as Arthropoda, there have been mitochondrial T-arm-lacking tRNA genes discovered (Masta, 2000). Interestingly, there are two mt EF-Tu genes in arthropods (Ohtsuki and Watanabe, 2007). The functional differences between the two EF-Tu homologs in these species should be elucidated, and this project is in progress in our laboratory.

## FUTURE PERSPECTIVES

Besides ARS and EF-Tu, other factors such as tRNA terminal nucleotidyltransferases (CCA enzymes) and ribosomes could be interesting in terms of their interactions with shrunken mt tRNAs.

After the trimming of 3′ extra sequences, 3′CCA sequences are added to 3′ ends of pre-tRNAs by CCA enzymes (Deutscher, 1983). The bacterial CCA enzyme binds to the acceptor-T helix of pre-tRNA (Tomita et al., 2004), and thus a T-arm-lacking tRNA precursor is not a good substrate for the bacterial CCA enzyme (Tomari et al., 2002). On the other hand, the chromadorean nematode *C. elegans* has two genes for CCA enzymes, one of which encodes a putative mt CCA enzyme (Tomari et al., 2002). The recombinant (putative) mt CCA enzyme of *C. elegans* can recognize and efficiently add a CCA sequence, not only to conventional cloverleaf tRNAs, but also to T- or D-arm-lacking tRNAs (Tomari et al., 2002). It would be interesting to know how the nematode mt enzyme recognizes T-arm-lacking mt tRNAs efficiently.

During translation, conventional bacterial tRNA interacts with several sites in the ribosome (reviewed by Khade and Joseph, 2010). In bacterial ribosomes, the T-arm of P-site tRNA interacts with ribosomal protein L5 (Korostelev et al., 2006; Selmer et al., 2006). At the A-site, the T-arm of tRNA interacts with ribosomal protein L16 (Selmer et al., 2006; Voorhees et al., 2009). The residues in the A-site finger (helix 69) of 23S rRNA interact with the D-arm of tRNA at the A- and P-sites (Korostelev et al., 2006; Selmer et al., 2006; Voorhees et al., 2009). In a structural model of *C. elegans* mt rRNAs, the corresponding rRNA positions exist (Mears et al., 2002). At the E-site, residues in the T- and D-loops interact with ribosomal protein L1 and helices 76, 77, and 78 in 23S rRNA (e.g., the L1 stalk; Korostelev et al., 2006; Selmer et al., 2006). Interestingly, the corresponding regions in nematode mt rRNA are missing (Mears et al., 2002). In general, mitochondrial ribosomal proteins are enlarged compared to their counterparts in bacteria (Koc et al., 2000; Suzuki et al., 2001), suggesting that mitochondrial ribosomal proteins may have alternate binding modes for truncated tRNAs. Further structural analysis of metazoan mt ribosomes (Sharma et al., 2003; Greber et al., 2014) would be helpful to reveal the detailed interaction mode between mt ribosomes and shrunken mt tRNAs.

Structural alterations of metazoan mt tRNAs have been compensated for by several interacting factors. The mode of compensation by these factors may explain why metazoan tRNAs have undergone truncation during evolution. Further investigation into the detailed binding modes between shrunken tRNAs and the interacting factors that co-evolved with them will shed light on how truncated tRNAs evolved.

## Conflict of Interest Statement

The authors declare that the research was conducted in the absence of any commercial or financial relationships that could be construed as a potential conflict of interest.
